# Rare presentation of inflammatory pseudotumour involving subcutaneous tissues with superficial fat sparing

**DOI:** 10.1259/bjrcr.20200154

**Published:** 2020-11-25

**Authors:** Domnique Newallo, Adam J Perricone, Anthony P Martinez, Dania Hussain, Saima Muzahir

**Affiliations:** 1 Division of Nuclear Medicine and Molecular Imaging, Department of Radiology and Imaging Sciences, Emory University, Atlanta, GA, USA; 2 Department of Pathology & Laboratory Medicine, Emory University, Atlanta, GA, USA

## Abstract

We present a unique case of inflammatory pseudotumour involving gluteal subcutaneous tissue with the sparing of superficial fat and report its contrast-enhanced CT, F-18 fluorodeoxyglucose positron emission tomography/CT and pathological findings. Although rare, inflammatory pseudotumours have been reported with a diverse spectrum of locations; however, the involvement of the subcutaneous tissue overlying the gluteal muscles with sparing of the most superficial fat has not been reported.

## Case presentation

Our patient is a 57-year-old male with no significant medical history who presented to the emergency department with chest pain at rest and shortness of breath. Additional history included unintentional 23 kg weight loss. On physical exam, right flank tenderness was noted, otherwise, the physical exam was unremarkable. Laboratory results showed anaemia with a haemoglobin of 6.8 gm dl^−1^ requiring blood transfusion. This prompted additional examination with a CT of the chest, abdomen and pelvis.

## Investigations

**Figure 1.**
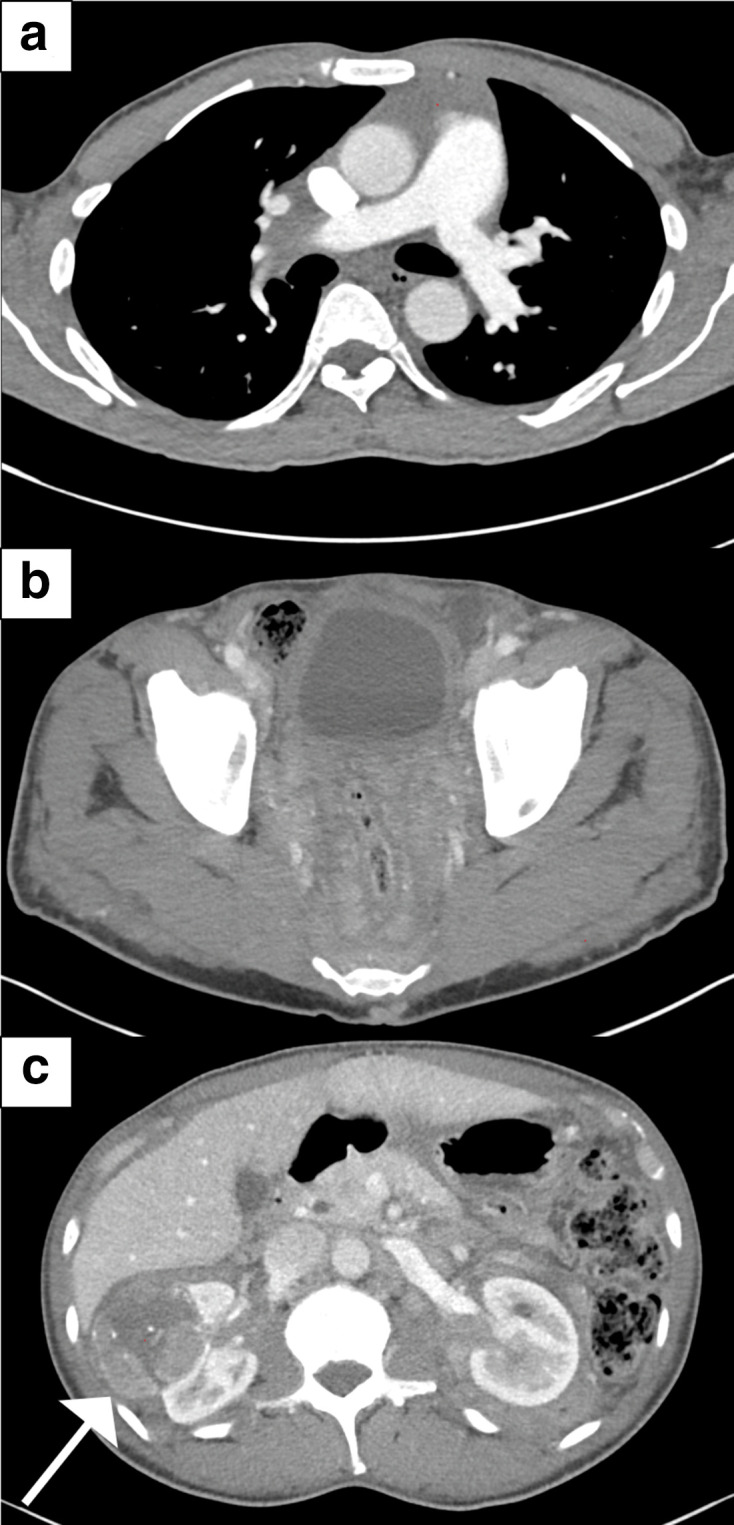
Axial images of the initial CT chest, abdomen and pelvis were remarkable for infiltrative soft-tissue density in the anterior mediastinum (a), retroperitoneum (b), gluteal deep subcutaneous fat (c) and mesorectal fat (c). Additional CT findings of the abdomen demonstrated a complex right renal mass found to be papillary renal cell carcinoma (white arrow).

CT imaging demonstrated a confluent soft-tissue density throughout the anterior chest (Figure 1a), retroperitoneum (Figure 1b), perirectal region (Figure 1c), gluteal (Figure 1c) and upper thigh subcutaneous fat with lymphoma as the leading differential consideration. Additionally, a complex right renal mass was noted along with right hilar and adjacent retroperitoneal lymphadenopathy. Given the imaging findings, a biopsy of the right renal mass and retroperitoneal soft-tissue density was recommended. Histopathological results from the renal mass core biopsy demonstrated a neoplastic proliferation of epithelial cells arranged in papillae, tubules and small solid nests, consistent with papillary renal cell carcinoma; concomitant immunohistochemical workup was supportive of this diagnosis. However, the biopsy of retroperitoneal soft-tissue confluence was non-diagnostic exhibiting benign fibroadipose tissue with fibrosis and a plasma cell-rich inflammatory process.

The case was presented at the institutional tumor board, and a decision was made to do an F-18 fluorodeoxyglucose positron emission tomography/CT (F-18 FDG PET/CT) to better evaluate the infiltrative soft-tissue in the chest, abdomen and pelvis as it was concerning for a second malignancy such as lymphoma.

**Figure 2.**
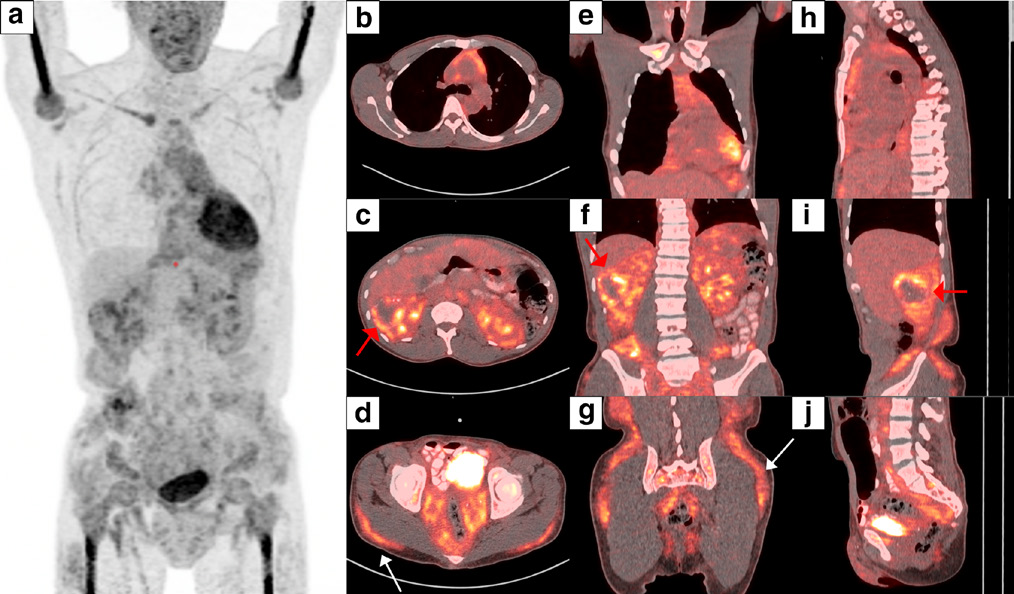
F-18 FDG PET CT. Maximum intensity projection image (a) demonstrating low-level metabolic activity (SUV_max_ up to 5) associated with diffuse infiltrative soft-tissue density in the mediastinum, abdomen and pelvis. Fused axial (b), coronal (e) and sagittal (h) images of the chest demonstrate low-level FDG uptake in the anterior mediastinum. Fused axial (c) coronal (f) and sagittal (i) images of the abdomen show low-level FDG avidity in the infiltrative soft-tissue of the retroperitoneum. Heterogeneous peripheral increased FDG uptake in the right renal mass (Red arrow). Fused axial (d), coronal (g) and sagittal (j) images of pelvis show FDG uptake in soft-tissue infiltrate in the mesorectal fat and deep subcutaneous fat overlying the gluteal muscles (White arrows).

F-18 FDG PET/CT was performed 60 min after the i.v. administration of 559 MBq of F-18 FDG on a GE Discovery 690 16-slice PET/CT scanner. PET/CT images showed low-level metabolic activity within the diffuse infiltrative soft-tissue involving the pericardiophrenic fat, retroperitoneum and mesorectal region, all illustrated in Figure 2. Additional low-level activity was noted to involve the deep subcutaneous tissues overlying the gluteal muscles with sparing of the superficial fat (Figure 2). Due to these findings, it was recommended to repeat the biopsy of an additional FDG avid tissue. The gluteal region was chosen as the next site of biopsy given the ease of access.

**Figure 3.**
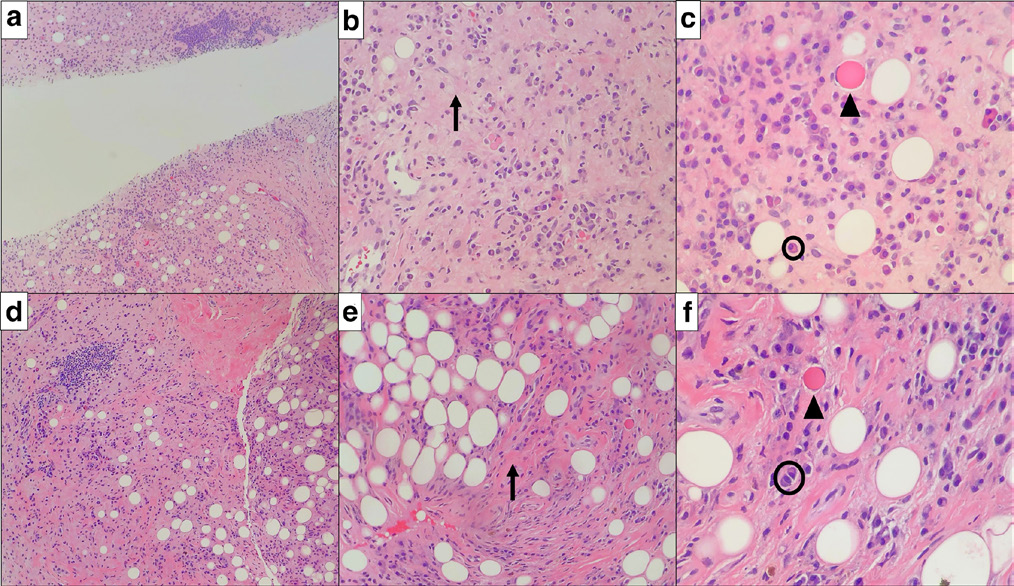
Representative histopathology from the retroperitoneal (a–c) and subcutaneous gluteal (d–f) lesions. Morphological features of each lesion are similar and demonstrate lymphoplasmacytic inflammation (representative plasma cells – black circles) and associated fine fibrosis and hyalinization (black arrows indicate representative hyalinized, fibrotic areas) of adipocytic soft-tissue. Scattered Russell bodies are seen (panels c and f; black arrowheads).

Histological findings of the subcutaneous gluteal region were essentially morphologically identical to the prior biopsy. Specifically, it demonstrated marked lymphoplasmacytic inflammatory infiltrate intracytoplasmic inclusions within a hyalinized, collagenous background. Focally, Russell bodies, eosinophilic intracytoplasmic inclusions of immunoglobulins within plasma cells, were seen. Intermixed with the inflammatory infiltrate were bland fibroblastic cells, with histiocytes only rarely present. Scattered thin-walled, dilated blood vessels were present, and no phlebitis was identified. Mitotic figures were sparse. The fibroinflammatory process was also seen interdigitating with focally necrotic adipose tissue, suggestive of at least microscopically infiltrative borders (Figure 3). Given the morphology, diagnostic considerations included inflammatory myofibroblastic tumour (IMT), IgG4-related disease, Rosai-Dorfman disease (RDD), mycobacterial pseudotumour, plasma cell dyscrasia, well-differentiated liposarcoma (WDLS) and inflammatory pseudotumour.

Ancillary histological studies were performed to help resolve the differential diagnosis. The aforementioned morphological findings, coupled with negative Anaplastic Lymphoma Kinase (ALK), immunohistochemistry rendered a diagnosis of IMT unlikely.^1–4^ Likewise, IgG4 immunohistochemistry revealed only rare positivity, ruling-out a diagnosis of IgG4-related disease.^4^ While S100 immunohistochemistry was positive, morphological features of RDD – large, epithelioid histiocytes with abundant cytoplasm and emperipolesis – were not appreciated.^5^ Special stains for acid-fast organisms were negative, excluding a diagnosis of mycobacterial pseudotumour. *In situ* hybridization for κ and λ light chains revealed polytypic plasma cells; furthermore, flow cytometry was performed on material from the retroperitoneal lesion and failed to identify any clonal hematolymphoid proliferations. Subsequently, fluorescent *in situ* hybridization and immunohistochemistry assessing for amplification of MDM2 each resulted in negative, precluding a diagnosis of WDLS.^6^ Together, the above morphology and lack of any specific findings on ancillary studies were most in keeping with a diagnosis of inflammatory pseudotumour.

## Treatment

Following an investigation with imaging and pathology, the patient was referred to rheumatology for further management. The presence of inflammatory pseudotumours is suspected of having relations with infection, inflammation, trauma or other malignancies. Given the supposed association with malignancy, it was determined that the presentation of inflammatory pseudotumour was likely resulting from the concomitant renal cell carcinoma. Treatment with high dose steroids was held, and the patient underwent an uncomplicated right nephrectomy.

## Outcome and follow-up

To our knowledge, superficial sparing of the subcutaneous tissue in the gluteal tissue in conjunction with inflammatory pseudotumour is an unreported finding. It was felt that the treatment of the patient’s primary malignancy might reduce the presence of inflammatory pseudotumour. Following an uncomplicated right nephrectomy, the patient will be scheduled for follow-up PET/CT imaging to determine if there is a need for further intervention or treatment of the inflammatory pseudotumour.

## Discussion

Inflammatory pseudotumour encompasses a group of non-neoplastic, reactive proliferations comprised of a variable number of fibroblasts and mononuclear inflammatory cells, which has been known to be mistaken for malignant lesions clinically and radiologically. Although a benign process, treatment consists of excision, irradiation, chemotherapy agents or high-dose steroids for unresectable lesions. These rarely recur or progress following complete surgical removal.^7,8^ The morphological findings are non-specific, and, consequently, it is a diagnosis of exclusion with reported associations of infection, inflammation, trauma or other malignancies.^7,9,10^ Furthermore, inflammatory pseudotumour has been described in the literature by many names, including inflammatory myofibroblastic tumour (IMT), calcifying fibrous tumour, fibrous pseudotumour, inflammatory myofibrohistiocytic proliferation and inflammatory fibrosarcoma, all of which demonstrates variable histological characteristics and behaviour of these lesions.^7,11^

IMT, the term that has been most used in literature in conjunction with inflammatory pseudotumours, which is reported to exhibit malignant transformation, is most commonly found in the lungs, orbit, or retroperitoneum with histology noting proliferation with prominent myofibroblastic spindle cells and histiocytes. It is noted to have varying levels of fibrosis, necrosis, and chronic inflammatory cells. At the molecular level, chromosomal translocations leading to activation of the ALK, a receptor tyrosine kinase suggesting a neoplastic cause, is observed in ~40–100% of IMT cases, depending on the anatomical sites at which they arise.^1–4,9^ While used interchangeably, it has been stated that there are differences between the widely reported IMT and inflammatory pseudotumours.^12^ The distinction between the two is widely controversial, with reports suspecting that some types of inflammatory pseudotumours and IMT may represent both ends of a spectrum of one entity and is beyond the scope of this report.^2,11^

Radiographically, inflammatory pseudotumours are non-specific, featuring variable degrees of locations and attenuation that can present as distinct masses or infiltrative tissue possibly due to the fluctuating degrees of fibrosis, cellular infiltration and dynamic change during the inflammatory process.^7,13–16^ When imaged FDG, there is uptake similar to malignancy due to the infiltrating inflammatory cell composition.^17,18^ Contrast-enhanced CT may show a homogeneous or heterogeneous lesion with low, equal or high attenuation compared with the surrounding tissue. Ultimately, the characteristics of the imaging findings depend on the site of origin of the lesion and histological composition.^9^ Multiple imaging findings have been reported in the literature of histologically confirmed IMT, although imaging of inflammatory pseudotumours, specifically without mention of IMT, has yet to be reported.

Diagnosis of inflammatory pseudotumours radiographically is challenging given they can mimic a malignant process with variable known anatomic sites. For example, reported appearance using F-18 FDG PET/CT in the liver, lung, mesentery, kidney and colon shows varied features of uptake intensity and distribution.^9,17,19^ Ultimately, for the diagnosis of inflammatory pseudotumour, multiple radiological studies should be compared, although an accurate finding will require histological examination. When a diagnosis of inflammatory pseudotumour or IMT is considered, surgery is considered the treatment of choice if it is resectable. However, it may be avoided or delayed if the extent of disease is monitored using F-18 FDG PET/CT.^8^ Other treatment options include irradiation, chemotherapy agents and high-dose steroids for unresectable lesions.^7,8^

In our case, CT images demonstrated a complex right renal mass (which was then biopsied and was found to be renal cancer) along with diffuse infiltrative soft-tissue involving the mediastinum, retroperitoneum and subcutaneous tissues. The FDG uptake was mild, although heterogeneous in appearance, which may be attributed to the varying characteristics of infiltrating inflammatory cell composition or fibrosis. The increased uptake likely reflects the activity of the inflammatory cells while the intervening fibrosis limits activity to be mild. Inflammatory pseudotumours centered in the soft-tissue usually displace and distort adjacent tissues, while in this case, there was no distortion in the surrounding subcutaneous tissues. Although rare, further knowledge of this entity can help in preventing overdiagnosis and aggressive treatment as a malignancy.

## Learning points

1. Inflammatory pseudotumour is an uncommon benign disease that can arise anywhere in the body with clinical and radiological appearance mimicking malignancy.
2. F-18 FDG shows a varying degree of uptake and is highly sensitive but not specific for the diagnosis of inflammatory pseudotumour. F-18 FDG PET/CT, however, could be useful in identifying the site for biopsy and monitoring treatment response in non-surgical candidates.
